# Field expansion of DNA polymerase chain reaction for early infant diagnosis of HIV-1: The Ethiopian experience

**DOI:** 10.4102/ajlm.v2i1.31

**Published:** 2013-05-22

**Authors:** Peter N. Fonjungo, Mulu Girma, Zenebe Melaku, Teferi Mekonen, Amilcar Tanuri, Bereket Hailegiorgis, Belete Tegbaru, Yohannes Mengistu, Aytenew Ashenafi, Wubshet Mamo, Tesfay Abreha, Gudetta Tibesso, Artur Ramos, Gonfa Ayana, Richard Freeman, John N. Nkengasong, Solomon Zewdu, Yenew Kebede, Almaz Abebe, Thomas A. Kenyon, Tsehaynesh Messele

**Affiliations:** 1 Center for Disease Control and Prevention (CDC), Addis Ababa, Ethiopia; 2 Ethiopian Health and Nutrition Research Institute (EHNRI), Addis Ababa, Ethiopia; 3 ICAP, Columbia University, Addis Ababa, Ethiopia; 4 John Hopkins University TSEHAI program, Addis Ababa, Ethiopia; 5 University of Washington, ITECH Program, Addis Ababa, Ethiopia; 6 Center for Global Health, Centers for Disease Control and Prevention (CDC), Atlanta, USA; 7 Clinton HIV/AIDS Access Initiative (CHAI), Addis Ababa, Ethiopia

## Abstract

**Background:**

Early diagnosis of infants infected with HIV (EID) and early initiation of treatment significantly reduces the rate of disease progression and mortality. One of the challenges to identification of HIV-1-infected infants is availability and/or access to quality molecular laboratory facilities which perform molecular virologic assays suitable for accurate identification of the HIV status of infants.

**Method:**

We conducted a joint site assessment and designed laboratories for the expansion of DNA polymerase chain reaction (PCR) testing based on dried blood spot (DBS) for EID in six regions of Ethiopia. Training of appropriate laboratory technologists and development of required documentation including standard operating procedures (SOPs) was carried out. The impact of the expansion of EID laboratories was assessed by the number of tests performed as well as the turn-around time.

**Results:**

DNA PCR for EID was introduced in 2008 in six regions. From April 2006 to April 2008, a total of 2848 infants had been tested centrally at the Ethiopian Health and Nutrition Research Institute (EHNRI) in Addis Ababa, and which was then the only laboratory with the capability to perform EID; 546 (19.2%) of the samples were positive. By November 2010, EHNRI and the six laboratories had tested an additional 16 985 HIV-exposed infants, of which 1915 (11.3%) were positive. The median turn-around time for test results was 14 days (range 14–21 days).

**Conclusion:**

Expansion of HIV DNA PCR testing facilities that can provide quality and reliable results is feasible in resource-limited settings. Regular supervision and monitoring for quality assurance of these laboratories is essential to maintain accuracy of testing.

## Introduction

The Joint United Nations Programme on HIV and/or AIDS (UNAIDS) has estimated that in 2009 between 230 000 and 510 000 children under the age of 15 years became infected with HIV worldwide, with over 90% of these new infections occurring in sub-Saharan Africa, and mainly through mother-to-child-transmission.^1^ Disease progression is aggressive in the first months of life in infants who acquired the infection *in utero* or around the time of delivery. If left untreated, almost half of these children will die before turning two years of age,^2^ and 75% by the age of five years. Most of these deaths in children with HIV could have been avoided through early infant diagnosis (EID) and provision of effective care and treatment. Interventions like the use of antiretroviral (ART) drugs by infected pregnant women, safe delivery practices and safe infant feeding have helped reduce the risk of transmission to infants (from 40% to 5–10%).^3,4,5,6^ Despite the availability of these life-saving interventions, some exposed infants still get infected.

In 2003, ART services were initiated in Ethiopia and by 2005 free ART services started with the support of the President’s Emergency Plan for AIDS Relief (PEPFAR) and the Global Fund to Fight AIDS, Tuberculosis and Malaria (GFATM).^7^ Furthermore, these services have been rolled out to regional health facilities at both hospital and health centre levels. The total number of patients on ART as of February 2010 was estimated at 179 183,^8^ including 10 496 children. One of the major challenges to expansion of ART to children is limited or inadequate HIV diagnostic capacity.

Early identification of infants infected with HIV followed by prompt ART treatment can help reduce morbidity and mortality. The need for early initiation of treatment in infants infected with HIV was emphasised in a South African cohort study that showed that 55% and 85% of HIV-infected infants had their CD4 cell count reduced to less than 25% by three and six months after birth, respectively.^9^ Another study conducted in South Africa showed that EID and early initiation of antiretroviral therapy helped to reduce mortality by 76% and HIV progression by 75%.^10^ Other studies have shown improved outcomes when early ART treatment is started in children.^11,12^ Thus, laboratory diagnosis plays a critical role in increasing access of HIV-exposed infants to testing, and eventual timely and effective treatment and care.

In adults, HIV is diagnosed by testing for the presence of antibodies to HIV in blood. In infants, antibody testing is inadequate, as persistent passively acquired maternal antibodies in the infant may yield false-positive results for up to 18 months or longer.^13,14^ It is important to provide accurate diagnostic services for identification of infants infected with HIV. Because of its high sensitivity and specificity, DNA polymerase chain reaction (PCR) has been widely used for diagnosis of HIV amongst exposed infants.^15,16,17,18^ The technology allows for PCR to be performed using a small spot from a dried blood spot (DBS) sample, as well as identification of infection from birth.^19^ Additionally, this molecular test has been successfully established in several resource-limited settings to increase access to treatment.^20,21,22,23^

Ethiopia has a tiered laboratory network, which is a hierarchical or ladder-like system with the national reference laboratory at the top followed by regional, referral and/or specialised hospital laboratories, then district and health centre laboratories. The tiered laboratory network reflects the structure through which health services are delivered to the population, and is under the coordination of the Ethiopian Health and Nutrition Research Institute (ENHRI), which is the technical arm of the Ministry of Health, with responsibility and oversight of the country’s public health laboratories. EHNRI has developed a comprehensive national laboratory master plan for integrated diseases. It has been used to coordinate and guide partners and stakeholders in the implementation of strategic laboratory objectives. The importance of the master plan in health system strengthening has previously been described.^24^

Until October 2007, EHNRI, which hosts Ethiopia’s national HIV reference laboratory, was the only facility performing DNA PCR for early infant diagnosis. EHNRI was able to serve the entire country with the implementation of a sample referral system which allowed for DBS to be transported and tested, and returned results to the different regions of the country. Ethiopia consists of about 1 127 127 square kilometers of land area, ranking it tenth in Africa in terms of land size.^25^ The challenge was to scale up laboratory capacity for DNA PCR in selected regions of Ethiopia in order to provide better and more adequate coverage.

Here we report on the experience of expansion of DNA PCR testing capabilities for early infant diagnosis to six different sites in Ethiopia with a substantial increase in the numbers of HIV-exposed infants tested.

## Materials and methods

### Project area

The project was carried out in Ethiopia, a country with a large land area situated in the horn of Africa and divided into nine administrative regions and two metropolitan city administrations, Addis Ababa and Dire Dawa. HIV prevalence is estimated at 2.1%.^26^ There are an estimated 64 813 children under the age of 14 years living with HIV and/or AIDS, 75 420 HIV positive pregnant women per year with an estimated 14 138 new infections occurring in children annually.^26^ The decentralisation of DNA PCR laboratories for EID was envisaged for six different sites to cover a greater geographic population.

### Approach to project implementation

In 2006, the national implementation plan for EID of HIV infection was developed by EHNRI with the support of the US Centers for Disease Control and Prevention (CDC) and its partner, Columbia University’s International Center for AIDS Care and Treatment Program (ICAP). The DBS-based DNA PCR technology using the Amplicor HIV-1 Monitor v1.5 manual assay (Roche Diagnostics, Indianapolis Indiana, USA) was evaluated and EID services established at the national HIV reference laboratory of EHNRI. DNA PCR testing of DBS specimens at the national HIV reference laboratory started in April 2006. Because of the vastness of the country and the time taken to transport DBS samples to the only EID laboratory at EHNRI, there was the need to decentralise DNA PCR testing for EID to the regions. In 2007, a joint assessment between CDC Ethiopia, EHNRI and university partners was conducted on the facilities in the identified regions. Prior to this, a site assessment checklist was developed and used to assess potential sites. The joint site visit assessed state of infrastructure, personnel capacity, water, electricity and inventory, and provided recommendations. Technical inputs were provided in the design of the laboratories, including physical separations of pre-PCR and post-PCR rooms. University partners (John Hopkins, Columbia University and University of Washington) refurbished the laboratories in six regions for carrying out DNA PCR for EID.

CDC Ethiopia and EHNRI developed and purchased a comprehensive list of laboratory equipment, reagents and consumables. All sites had generators to serve as back-up for power outages. Standard operating procedures were developed and distributed to all six sites. Training materials were developed by EHNRI in collaboration with CDC Ethiopia and ICAP-Columbia University and used at each site. At least two laboratory technologists were trained per site on DNA PCR for EID using DBS. Following training, laboratory technologists were independently assessed for accurately performing the test. Two methods were used for assessing staff competency: the direct observational method was employed where the technologists were closely observed by a mentor as they performed assays independently per the SOPs and corrective actions provided where needed. Additionally, each technologist was provided with six DBS of known results (blinded DBS specimen) and asked to perform assays unsupervised. As part of maintaining quality of testing in each laboratory, yearly refresher trainings were conducted, followed by challenging trainees with blind DBS specimens. Additional trainings were provided for selected facilities based on the needs identified. Also, to control for quality of testing in the laboratory, any two or more consecutive Enzyme-linked immunosorbent assay (ELISA) wells samples that were positive were each repeated from the same DBS card sample on a different day. EHNRI served as a quality assurance laboratory for retesting the first 100 DBS specimens for each of the newly established sites.

**FIGURE 1:**
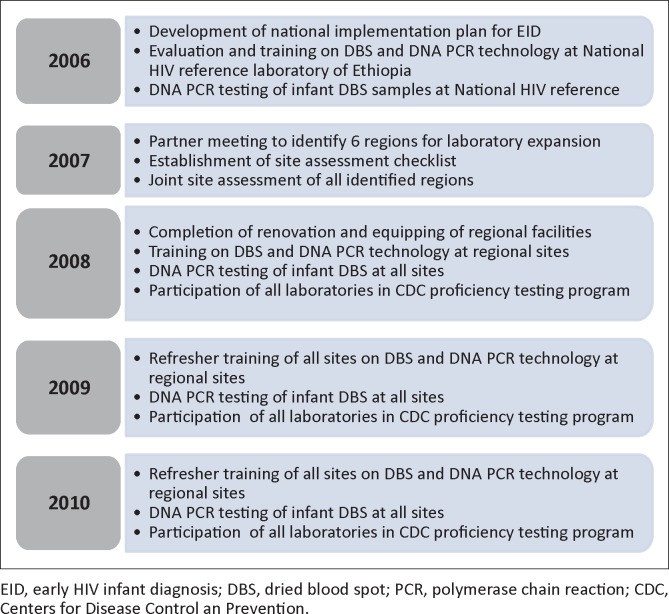
Milestones in expansion of laboratory services for early HIV infant diagnosis in Ethiopia.

A comprehensive EID supply list was developed and laboratory technologists were trained in inventory management to ensure availability of supplies and thus avoid disruption of services as a result of shortage of one or several commodities. DNA PCR testing started at the Addis Ababa regional laboratory in April 2008, and at the Mekelle, Adama and Harar regional laboratories in May 2008, whilst at Bahir Dar and Hawassa it started in June 2008. Analysis of DBS tested for all sites was done up to November 2010. All six laboratories were enrolled in the CDC Atlanta external quality assessment programme to monitor laboratory performance for DNA PCR testing. Proficiency testing (PT) panels were sent three times per year to each testing site. Significant stages in the development and expansion of laboratory services for DNA PCR for EID are presented here (Figure 1).

In 2010, the postal service was introduced to transport DBS samples from referring sites to EID laboratories as well as delivering results back to referring sites. Prior to initiation of sample referral using the postal service, a memorandum of understanding was signed between the Ethiopian Postal Services Enterprise and EHNRI that included 565 facilities referring infant DBS samples. Postal workers were trained in transportation of DBS, filling in of DBS samples transport log forms indicating date and time of pick up and drop off from DBS referring sites and EID laboratories, respectively. Before the implementation of postal services in 2010, DBS samples and results were transported by an assigned facility staff member and/or university partners.

The laboratory turn-around time was established with the help of the EID laboratory logbook. Each DBS sample collected from an infant was accompanied by an EID request form from the referring site. The laboratory technologists recorded in the EID laboratory log book the date the DBS sample was received at the laboratory. When EID results are ready, the date the results are released or returned from the laboratory is recorded and signed for by a university partner or postal worker for the return of results to the referring site.

## Results

### Laboratory infrastructure and training

**FIGURE 2:**
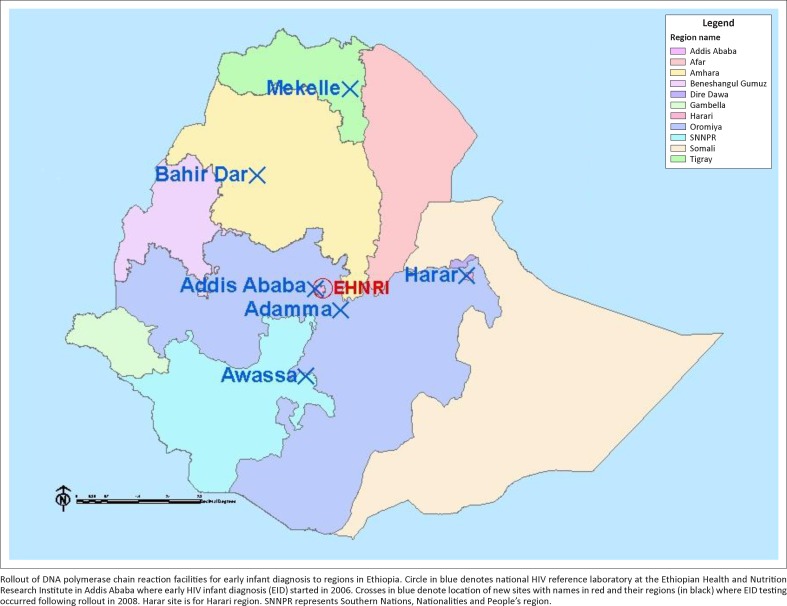
Regionalisation of early infant diagnosis

The six regional laboratories identified for expansion were strategically located around the country and included (1) Bahir Dar for the Amhara region, (2) Mekelle for Tigray region, (3) Hawassa for the Southern Nations, Nationalities and People’s (SNNP) region, (4) Adama for the Oromia region, (5) Harar for the Harari and (6) Somali regions and Addis Ababa for Addis Ababa city administration (Figure 2). By June 2008, all six identified regional reference laboratories had been completely renovated and well equipped with the instruments, software and laboratory consumables required for performing HIV-1 DNA PCR. The laboratory technologists were trained in laboratory biosafety, DBS specimen reception and/or rejection using criteria developed by CDC,^27^ accurately performing and interpreting test results, and reporting results back to referral sites. A total of 12 laboratory technologists (2 per site) were successfully trained and certified competent on-site for HIV-1 DNA PCR testing. The laboratory technologists each scored 100% in their competency assessment when challenged with six blind specimens. That is, they correctly interpreted test kit controls, in-house controls, as well as all blind DBS specimens. The modest number of laboratory technologists trained was aimed at ensuring that testing services would be minimally disrupted should one technologist be absent. Sample referral testing documentation and mechanisms to enable specimens to be transferred from referring sites to laboratories performing DNA PCR as well as reporting results back to referring sites were fully established.

### Scale-up of laboratory services

**FIGURE 3:**
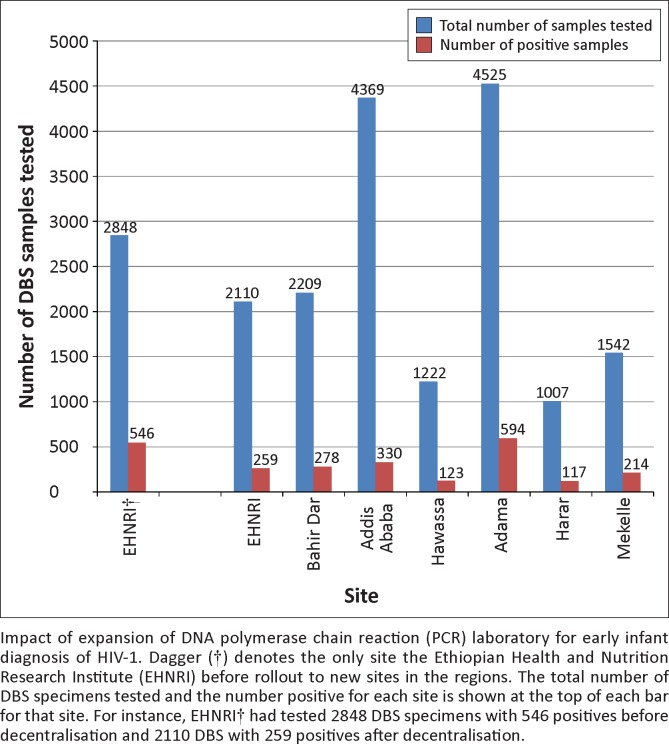
Proportion of dried blood spot (DBS) tested positive by site.

Prior to April 2008, only prior to decentralisation the national HIV reference laboratory at EHNRI, located in Addis Ababa, was performing DNA PCR for EID (Figure 2). From 2006 to April 2008, the national HIV reference laboratory had tested a total of 2848 infants’ DBS samples, of which 546 (19.2%) were positive and the results were sent back to referring sites (Figure 3). The DBS of HIV-exposed infants from prevention of mother-to-child transmission services and diagnostic specimens for clinical symptoms were tested. The results obtained for all laboratories that tested and provided infant DBS results up to November 2010 are shown in Figure 3. Of the 2209 tested for Bahir Dar, 278 (12.6%) were positive; 330 (7.5%) of 4369 were positive for Addis Ababa; 123 (10.1%) of 1222 were positive for Hawassa; 594 (13.1%) of 4525 positive for Adama; 117 (11.6%) of 1007 tested positive for Harar; and 214 (13.9%) of 1542 tested positive for Mekelle (Figure 3). Following decentralisation, EHNRI tested 2110 specimens, of which 259 (12.3%) were positive (Figure 3, EHNRI).

**FIGURE 4:**
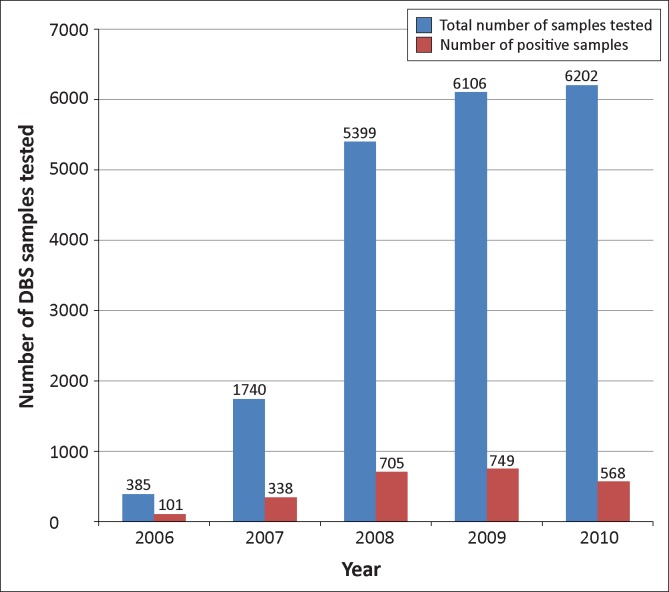
The total number of dried blood spot (DBS) specimens tested and the number positive by year between 2006 and 2010.

Although the number of specimens tested at EHNRI declined, EHNRI was still serving as a quality assurance laboratory for the newly established sites and testing specimens received from non-governmental organisation facilities, military and police hospitals, Afar, Gambella and Benishangul Gumuz regions. Of the total 19 832 DBS specimens tested from all testing sites between April 2006 and November 2010, 2461 (12.4%) were positive. At the end of 2007, 26 clinical sites were collecting DBS samples. A total of 565 active facilities are currently sending infant samples to these testing laboratories. Together with the establishment of these new collection sites, there was a substantial increase in the number of samples tested following decentralisation of laboratory services from 2008 and each year thereafter (Figure 4).

Before decentralisation (April 2006 to March 2008), all the DBS samples were tested only at EHNRI laboratory and the median turn-around time was 13 days (range 1–59 days). After decentralisation (April 2008 to November 2010), the median turn-around time at EHNRI was 12 days (range 1-63 days). After decentralisation, the other six regional laboratories in the country had a median turn-around time of 14 days (range 14–21 days).

### Quality of DBS DNA PCR testing

In addition to routinely conducting CDC Atlanta ’in-house’ DBS controls and test kit controls to validate performance of assays, all laboratories performed satisfactorily by scoring 100% in all PT sessions with the CDC PT panel programme. The quality assurance results on the first 100 DBS specimens for each of the newly established sites was concordance between results of EHNRI and those of the newly established laboratories. The repeat testing of any two or more consecutive positive samples showed concordance between the initial and the repeated test results.

## Ethical considerations

Because this was a programme impact assessment of expansion of DNA PCR laboratory for EID, no ethical approval was required.

## Discussion

In this project, we have demonstrated the feasibility of laboratory expansion of DNA PCR testing for EID in a resource-limited setting. Following the expansion of testing facilities and the establishment of new collection sites, there was an increase in the number of infant DBS samples tested. Early diagnosis of HIV-infected infants can enable faster access to treatment and, in combination with other indicators, can contribute to monitoring the success in programmes for the prevention of mother-to-child transmission. HIV DNA PCR is recommended as the gold standard for virologic diagnosis of HIV in infants. However, issues with the high expense associated with establishing a virologic testing laboratory, technical complexities as well as the need to ensure good quality control and assurance in a resource-constrained setting have been raised. This has led to the establishment of a few facilities in centralised areas in resource-limited countries.

The commitment of the government and partners was key to the rapid and successful regional expansion of EID molecular technology. The expansion of the laboratory diagnostics services is in line with one of the strategic objectives of the master plan developed by EHNRI. This further demonstrates the importance of developing a master plan with clear strategic objectives that serves as a rallying and coordinating tool for implementing laboratory programmes amongst several laboratory stakeholders as well as evaluation of the implementation of the master plan. There was also the need to quickly establish good laboratory practice in these new facilities and to maintain quality of testing, considering the technical complexities of such molecular methods and the fact that infants may only have the opportunity for a single HIV test to determine their HIV infection status. Proper on-site training and competency assessment coupled with yearly refresher training and regular joint supportive site visits by EHNRI and partners have been important to maintaining quality testing. This has also been reflected in the satisfactory performance (100%) of all the facilities in CDC external quality assessment through PT panels. Following decentralisation of DNA PCR testing sites and scaling up of the number of collection sites, we observed a significant increase in the number of infants tested with DBS without an increase in the turn-around time from when specimens are received at the laboratory to return of results from the laboratory. With decentralisation, we observed that turn-around time of results to referring sites was a median of 14 days. This reflects a strong and functional sample and results referral system for expediting results. However, because of the low number of DBS specimens and the need for batch testing, it still took Harar and Hawassa up to three weeks to return results.

WHO guidelines recommend that HIV virologic tests be used to diagnose HIV infection in infants and children up to the age of 18 months.^29^ Additionally, the guidelines emphasise post-natal follow up of infants with unknown or uncertain HIV exposure in order to have their HIV exposure status ascertained and that HIV-infected infants be put immediately on ART. Although 90% of children living with HIV acquired their infection through mother-to-child transmission, relatively few have access to testing, treatment and care.^30^ There are several challenges to implementing early treatment for HIV-infected infants, including the diagnosis of HIV-infected infants, lack of rapid diagnostic capabilities for infants, weak sample referral testing systems, under-developed quality controls, limited trained personnel, inadequate clinical systems to follow up with patients, lack of integration amongst health services, and exogenous social factors. Addressing these challenges is likely to improve and maintain quality of testing and lead to increase uptake of HIV-infected infants to receiving treatment.

Direct comparison of the number of DBS tested at the different sites is difficult. The number of infants tested at the new facilities varied greatly, with Bahir Dar, Addis Ababa and Adama each having tested about two or more times as many as Hawassa or Harar. The observed difference could partly be explained by the population size and HIV prevalence of these regions, as well as the robustness of their PMTCT programme and clinical practices. Of the nine regions, Bahir Dar and Adama in Amhara and Oromiya regions, respectively, account for 23% and 36%, respectively, of the general population. Also, the HIV prevalence of the two regions is 2.7% and 1.5% for Amhara and Oromiya, respectively.^26^ The city of Addis Ababa site also showed an increase in DBS testing but accounts for only 3% of the population. However, the city of Addis Ababa has a relatively higher HIV prevalence of 7.5%.^26^ By contrast, Hawassa in the SNNP region, which accounts for about 20% of the population, tested only 1222 DBS specimens. Together with the Harar and Mekelle sites, they tested fewer DBS specimens. These low numbers could be due to challenges with coordination of pediatric HIV care and treatment, PMTCT, and laboratory services in these regions.

Despite the successful scale-up of laboratory diagnostic services, significant challenges still persist and pose a problem for the provision of more comprehensive services for diagnosis and treatment of children. For example, the need for proper integration or linkages between programmes (e.g., laboratory and clinicians), identification of pregnant women, enrolling and scaling-up of the PMTCT programme. Furthermore, it has been difficult to determine the number of infants testing positive who received their test results and who received treatment and care. It is important to put in place a system for tracking DBS-positive results to ensure those infants receive treatment as well as for negative PCR results, to ensure that uninfected infants who continue to be exposed to HIV receive a final diagnosis. Similarly, improving the system of follow-up for infants and their caretakers is critical to avoid delays in initiating treatment.

The rapid scale-up of laboratory services to perform DNA PCR for EID and treatment of infected children is an essential component to child survival and successful outcomes of PMTCT programmes. Proper joint planning, implementation, commitment and coordination involving stakeholders have made rapid scale-up possible. Continuous monitoring of these new sites is essential, and adhering to all aspects of quality would ensure that accurate results are provided for prompt intervention.
